# Biomarker profiles of Alzheimer’s disease and dynamic of the association between cerebrospinal fluid levels of β-amyloid peptide and tau

**DOI:** 10.1371/journal.pone.0217026

**Published:** 2019-05-14

**Authors:** Aysha S. Mohamed Lafirdeen, Emmanuel Cognat, Severine Sabia, Claire Hourregue, Matthieu Lilamand, Aline Dugravot, Elodie Bouaziz-Amar, Jean-Louis Laplanche, Jacques Hugon, Archana Singh-Manoux, Claire Paquet, Julien Dumurgier

**Affiliations:** 1 Cognitive Neurology Center, Saint Louis—Lariboisiere—Fernand Widal Hospital, AP-HP, Université de Paris, Paris, France; 2 Inserm U1153, CRESS, Epidemiology of Ageing and Neurodegenerative diseases, Université de Paris, Paris, France; 3 Department of biochemistry, Saint Louis -Lariboisiere—Fernand Widal Hospital, AP-HP, Paris, France; 4 Department of Epidemiology and Public Health, University College London, United Kingdom; Banner Alzheimer's Institute, UNITED STATES

## Abstract

**Objective:**

To investigate the relationship between cerebrospinal fluid (CSF) β-amyloid peptide (Aβ42) and CSF Tau in a large population of patients referred to memory clinics for investigation of cognitive dysfunction.

**Methods:**

We analyzed Alzheimer’s disease (AD) biomarkers in CSF taken from 3565 patients referred to 18 French memory clinics. Patients were classified into four profiles according to levels of CSF biomarkers (A: amyloidosis, N: neurodegeneration). The association between CSF Tau and CSF Aβ42 were analyzed using general linear regression models, in the overall population and stratified by biomarkers profiles. We compared linear and quadratic models using Akaike information criterion. We also assessed change in biomarker profiles in a subset of patients who had 2 assessments of biomarkers.

**Results:**

CSF Tau was negatively associated with CSF Aβ42 in the overall population, following a non-linear quadratic model. However, the nature of this association was different in the 4 profiles: positive association in A-N- profile, negative association in A-N+ and A+N+ profiles, lack of association in A+N- patients. When considering patients with longitudinal data on profiles, 36% of those initially classified as A-N+ evolved to an A+N+ profile.

**Conclusions:**

The nature of the association between CSF Aβ42 and CFS Tau depends on the A/N profiles of patients. These results suggest an increase in CSF Aβ42 early in the disease before its decline while tau pathology progresses, this pattern is particularly observed in non-APOE4 subjects. This phenomenon may explain why some patients with neurodegeneration only markers convert to an AD profile (A+N+) over time.

## Introduction

Alzheimer’s disease (AD), the most common cause of dementia, is neuropathologically characterized by extracellular β-amyloid peptide (Aβ42) deposits, neurofibrillary tangles composed of intraneuronal abnormally phosphorylated Tau, and neuronal and synaptic loss [1]. According to the “amyloid cascade” hypothesis [2], extracellular accumulation of Aβ is the first step, successively followed by deposits of neurofibrillary tangles, neuronal death and finally the clinical onset of symptoms [3]. A competing hypothesis is that neurofibrillary tangles appear first, particularly in sporadic AD [4, 5].

The development of AD biomarkers during the past decade allows new opportunities for studying the in-vivo kinetics of amyloid and tau lesions [6, 7]. Cerebrospinal fluid (CSF) measurement of Aβ42 has been shown to be inversely correlated with total brain Aβ load [8], while CSF tau levels positively correlated with the presence of neocortical neurofibrillary tangles [9]. Based on the amyloid hypothesis, a temporal chronology of AD biomarkers has been proposed [10], and in 2011 the National Institute on Aging-Alzheimer’s Association (NIA-AA) proposed biomarker based recommendations for the staging of preclinical AD [11]. Using evidence of amyloidosis (A) and neurodegeneration (N), Stage 1 corresponds to presence of Aβ deposits and the absence of neurodegeneration (A+N-), while stage 2/3 corresponds presence of both markers (A+N+), without or with subtle cognitive decline. One year after the publication of the NIA-AA recommendations, Jack et al. reported an additional category of subjects, characterized by positivity for only neurodegeneration markers (A-N+) and proposed the designation of SNAP, for Suspected Non-Alzheimer’s disease Pathophysiology for these subjects [12].

The concept of SNAP (A-N+) remains the subject of debate [13]. Several studies have shown accelerated cognitive decline in these persons [14–17], with poorer clinical prognosis than A+N- subjects [18]. However, other studies found no differences between SNAP and A-N- subjects in cognitive decline, while the A+N- and A+N+ groups declined faster [19–21]. It is worth noting that the precise biomarkers used to define SNAP remains heterogeneous across studies, some use CSF Tau or phosphorylated Tau, or T1-weighted structural MRI, and/or FDG PET imaging data.

CSF allows assessment of both Aβ42 and Tau, the hallmarks of AD, but their relationship remains poorly understood. A recent study based on data on 766 subjects from 3 independent cohorts found a non-linear quadratic association between CSF Tau and Aβ42 [22]. In the present study, we aim to extend these analyses using data on 3,565 patients in order to cover a wide spectrum of AD pathology. We test the hypothesis that the nature of the association between these biomarkers depends on the AD “profile” of patients. In further analysis on patients who had 2 lumbar punctures we analyzed the longitudinal change in biomarkers profiles to test the hypothesis that persons initially classified as SNAP may evolve to an AD profile.

## Methods

### Subjects

Following national recommendations, CSF analyses are routinely undertaken in patients with cognitive signs referred to hospital-based memory centers in France [23, 24]. For the present study, CSF biomarkers from 18 hospitals in and around Paris, France, were aggregated in one biochemistry department. All patients seen between January 1^st^ 2008 and January 1^st^ 2018 for whom AD biomarkers from CSF were available were included in the analysis; methods and characteristics of the population have been previously reported elsewhere [25, 26]. The study was approved by the Ethical Committee of Paris University Hospitals. Patients under legal protection were excluded. Written consent was signed in priority by the patients, and in case of inability to sign, by their caregivers. The consent procedure was approved by the local ethical committee.

### CSF analysis

Lumbar puncture was performed on patients in a fasting state, typically between 9 and 12 am. All centers used the same model of 10 mL polypropylene tube to collect CSF (January 2008 to November 2012: CML model TC10PCS; December 2012 to January 2018: Sarstedt catalog no. 62.610.201). CSF samples were centrifuged at 1,000 g for 10 minutes at 4°C within 4 hours of collection, and then aliquoted in 0.5 mL polypropylene tubes and stored at -80°C for further analysis. CSF levels of Aβ42, total Tau, and pTau-181 were measured with the commercially available sandwich ELISA INNOTEST, using the manufacturer’s procedures (Fujirebio Europe NV, formely Innogenetics NV). The Alzheimer’s Association quality control program for CSF biomarkers validated the quality of CSF evaluations of the hospital biochemistry department [27].

### Profiles based on CSF biomarkers

The CSF biomarker profiles were used to determine the “profile” of every subject using the NIA-AA staging framework [11]. The amyloidosis profile (A+N-) was characterized by a low level of CSF Aβ42 and normal levels of CSF Tau and CSF p-Tau 181. The amyloidosis combined with neurodegeneration profile (A+N+) was defined by a low level of CSF Aβ42 and increased level of CSF Tau and/or CSF p-Tau 181. Subjects with high levels of CSF Tau and/or CSF p-Tau 181 but normal Aβ42 were classified as SNAP (A-N+). Those with normal levels of all three biomarkers were classified as “normal” (A-N-).

In this study we chose not to use the recently proposed A/T/N classification[18, 28] as we have previously showed that some profiles (A-T+N-, A+T+N-) were quite rare, representing less than 1% of the sample [25]. Furthermore, in our data CSF Tau and CSF p-Tau 181 were highly correlated (Spearman coefficient correlation = 0.84, P<0.001).

Abnormal values of CSF biomarkers were defined using thresholds used routinely in clinical practice based on higher Youden index in order to discriminate AD patients from non-AD patients (i.e. best compromise for sensitivity and specificity). The type of the polypropylene tube changed in November 2012, and the ELISA INNOTEST sandwich changed on May 1^st^ 2016, requiring adjustment of thresholds at these 2 dates. The cut-offs for CSF Aβ42, Tau and p-Tau 181 moved respectively from 500/300/65 pg/mL [29] to 730/300/58 pg/mL [25] after the change of tube.

### Covariates

These included age, sex, and performance on the 30-item Mini Mental State Examination (MMSE) for an assessment of general cognitive status. APOEe4 status was known for a sub-sample of 1034 individuals.

### Statistical analysis

Participant characteristics were presented overall and by profiles defined using NIA-AA CSF biomarkers (A-N-, A+N-, A+N+, A-N+). Proportions were calculated for categorical variables, while means and standard deviations were computed for continuous variables. Differences as a function of profiles were assessed using a χ^2^ test or Student t-test as appropriate.

The association between CSF Tau and Aβ42 was examined using general linear regression models with CSF Aβ42 as the dependent variable (PROC GLM of SAS). The analysis was initially adjusted only for the type of collection tube (unadjusted model), and then further adjusted for age, sex, and APOEe4 status. Analysis were first undertaken in the total sample and then stratified by the 4 profiles (A-N-, A+N-, A+N+, A-N+). The difference in the association between CSF Tau and Aβ42 in the 4 profiles was tested formally using interaction terms in the linear regression models.

We compared the linear model (Aβ42 = α + β xTau) to the quadratic model (Aβ42 = α + β xTau + γ x Tau^2^) following the Akaike information criterion (AIC), with lower AIC values designating better model fit [30]. Linear and quadratic models were compared in the overall population and then separately in each profile (A-N-, A+N-, A+N+, A-N+).

In the sub-sample with APOEe4 data (N = 1034) we examined the association between CSF Aβ42 and CSF Tau by first plotting the mean values of CSF Aβ42 as a function of deciles of CSF Tau in those with an APOEe4 allele versus none. These associations were also examined in linear regression analysis, adjusted for collection tube, age and sex, in two groups based on median CSF Tau.

Finally, in persons with 2 lumbar punctures with CSF AD biomarkers, we examined the evolution of their biomarkers profiles to assess conversion in stage of AD pathology.

The CSF biomarkers were not normally distributed and we followed the approach used by other teams by log-transforming CSF Tau and using CSF Aβ42 without any transformation in the statistical models [22]. CSF Tau and CSF phosphorylated Tau were highly correlated in our sample leading us to use CSF Tau in the main analyses; use of CSF p-Tau 181 in place of CSF Tau led to similar findings and conclusions.

All p-values were two-tailed and p≤0.05 was considered statistically significant. Statistical analyses were performed using SAS version 9.3 (SAS Institute, Cary, NC, USA).

## Results

CSF biomarker data were available on 3565 persons, assessed between January 1st, 2008 and January 1st, 2018 in one of the 18 memory clinics included in the study. Their characteristics are summarized in Table 1. The mean (SD) age was 69.8 (10.3) years, 50% were women, and the mean (SD) MMSE at the time of the lumbar puncture was 21.7 (6.0). Of the 3565 persons in the sample, 27% had a normal biomarker profile (A-N-), 36% had an AD profile (A+N+), 22% were positive only for amyloid (A+N-), and 15% had the SNAP profile—positive only for neurodegeneration (A-N+). Compared to the normal profile, patients with an AD profile were older, more often women, had a lower MMSE scores and were more likely to carry at least one APOE ε4 allele.

**Table 1:** Characteristics of the study population, overall and as a function of profiles defined by CSF biomarkers. Profiles are based on CSF Aβ42, CSF Tau, and CSF p-Tau (NIA-AA classification): A-N-: normal levels of all three biomarkers; A+N-: low level of CSF Aβ42 and normal levels of CSF Tau and CSF p-Tau 181; A+N+: lowe level of CSF Aβ42 and increased level of CSF Tau and/or CSF p-Tau 181; A-N+: high CSF Tau and/or CSF p-Tau 181 but normal Aβ42.

		Profiles defined by CSF biomarkers				
	Overall	A-N-	A+N-	A+N+	A-N+	
Characteristics	(N = 3565)	(N = 947)	(N = 789)	(N = 1299)	(N = 527)	P value
Age, years, mean (SD)	69.8 (10.3)	66.0 (11.1)	69.9 (10.5)	72.2 (9.1)	70.7 (9.7)	<0.001
Women, n (%)	1770 (49.7)	437 (46.2)	348 (44.1)	739 (56.9)	246 (46.7)	<0.001
MMSE, mean (SD)	21.7 (6.0)	23.7 (5.1)	21.6 (6.2)	20.0 (6.2)	22.5 (5.1)	<0.001
Education levels, years, mean (SD)	10.1 (4.5)	10.1 (4.6)	10.2 (4.6)	10.0 (4.4)	9.9 (4.3)	0.78
APOEε4 carriers, n (%)^a^	430 (41.6)	61 (19.2)	57 (50.4)	267 (60.5)	45 (27.8)	<0.001
CSF biomarkers (pg/ml), Mean (SD)						
Aβ42	682.1 (291.4)	942.1 (198.8)	517.7 (161.4)	479.6 (138.1)	961.3 (275.2)	<0.001
Tau	404.9 (326.3)	199.2 (56.6)	179.7 (67.8)	645.3 (344.6)	519.1 (327.2)	<0.001
p-Tau 181	60.4 (36.0)	38.8 (10.6)	33.0 (12.1)	88.5 (38.4)	70.9 (26.6)	<0.001

^a^APOE genotype determined in a sub sample of 1034 patients.

There was an inverse association between CSF Tau and CSF Aβ42 when the study population was considered together (β[SE] = -82.1[6.7], p<0.001, Table 2). In analysis stratified by profiles, CSF Tau was positively associated with CSF Aβ42 in A-N- subjects (β[SE] = 153.6[17.7]), and negatively associated with CSF Aβ42 among A+N+ (β[SE] = -19.7[7.2]) and A-N+ subjects (-125.2[22.9]), while no significant association was observed in A+N- patients. These associations were robust to adjustment for age, sex, and APOE4 status. The test for interaction was highly significant (<0.001) suggesting that the association between CSF Aβ42 and CSF Tau differed in the four profile groups.

**Table 2:** Association between CSF Tau (log transformed) and CSF Aβ42, linear regression analysis. Profiles are based on CSF Aβ42, CSF Tau, and CSF p-Tau (NIA-AA classification).

		Unadjusted^a^		Adjusted^b^	
Population	N	β(SE)	P	β(SE)	P
Overall	3565	-82.1 (6.7)	<0.001	-65.1 (6.7)	<0.001
Profiles					
A-N-	947	153.6 (17.7)	<0.001	178.5 (17.6)	<0.001
A+N-	789	18.7 (11.9)	0.12	22.9 (12.1)	0.06
A+N+	1299	-19.7 (7.2)	0.006	-20.0 (7.1)	0.005
A-N+	527	-125.2 (22.9)	<0.001	-116.5 (22.5)	<0.001

^a^Adjusted for nature of the tube.

^b^Adjusted for the tube, age, gender, APOE4 status.

**Fig 1:**
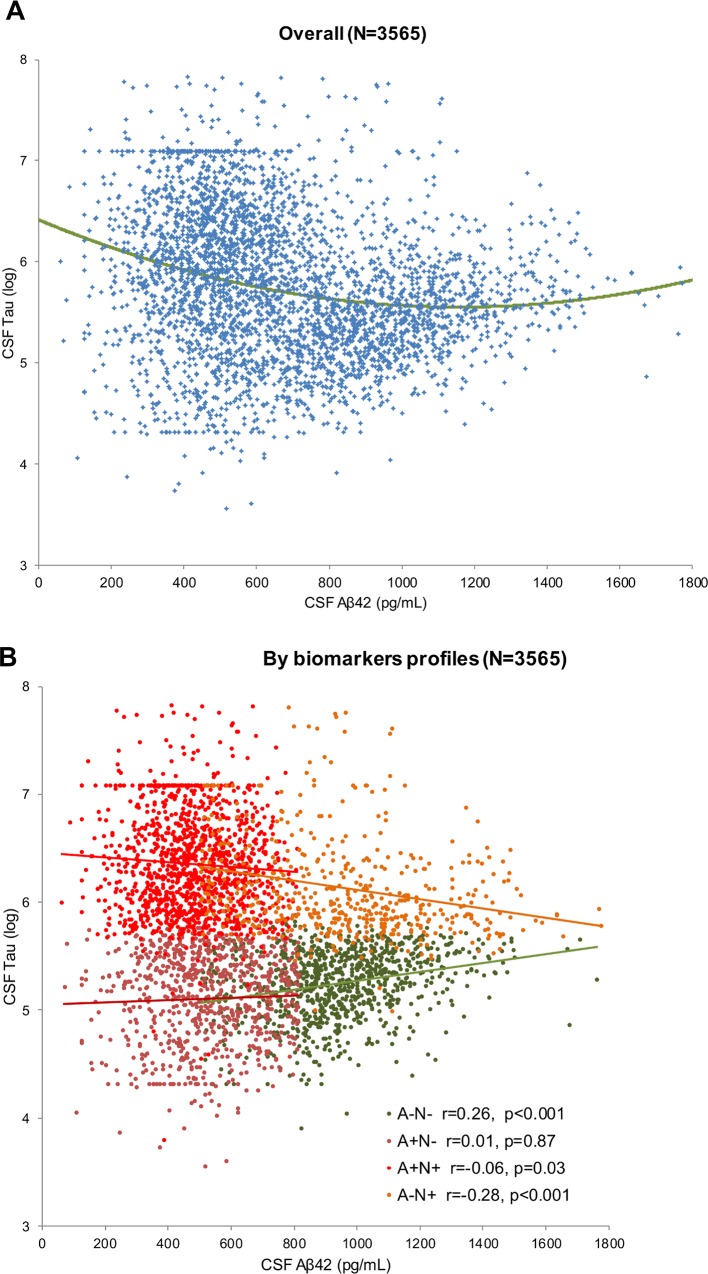
Scatter plot of CSF Aβ42 and log transformed CSF Tau. **A.** In the total study population. The quadratic model is presented in green. B. In analysis separately in each profile. Green: A-N-, brown: A+N-, orange: A-N+, red: A+N+. Linear regression models are presented and Spearman correlation coefficients.

Comparisons of linear and quadratic models for the association between CSF Tau and CSF Aβ42 are presented in S1 Table. The quadratic model fitted the data better in the total study population (delta AIC = 43). However, this was not the case in all profile sub-groups as the quadratic model was inferior to the linear model for A-N-, A+N- and A+N+ profiles (delta AIC = -2), and was only better than the linear model in A-N+ patients (delta AIC = 8). The scatter plot of CSF Aβ42 and CSF Tau is presented in Fig 1, in the total study population and in the profiles defined by biomarkers.

**Fig 2:**
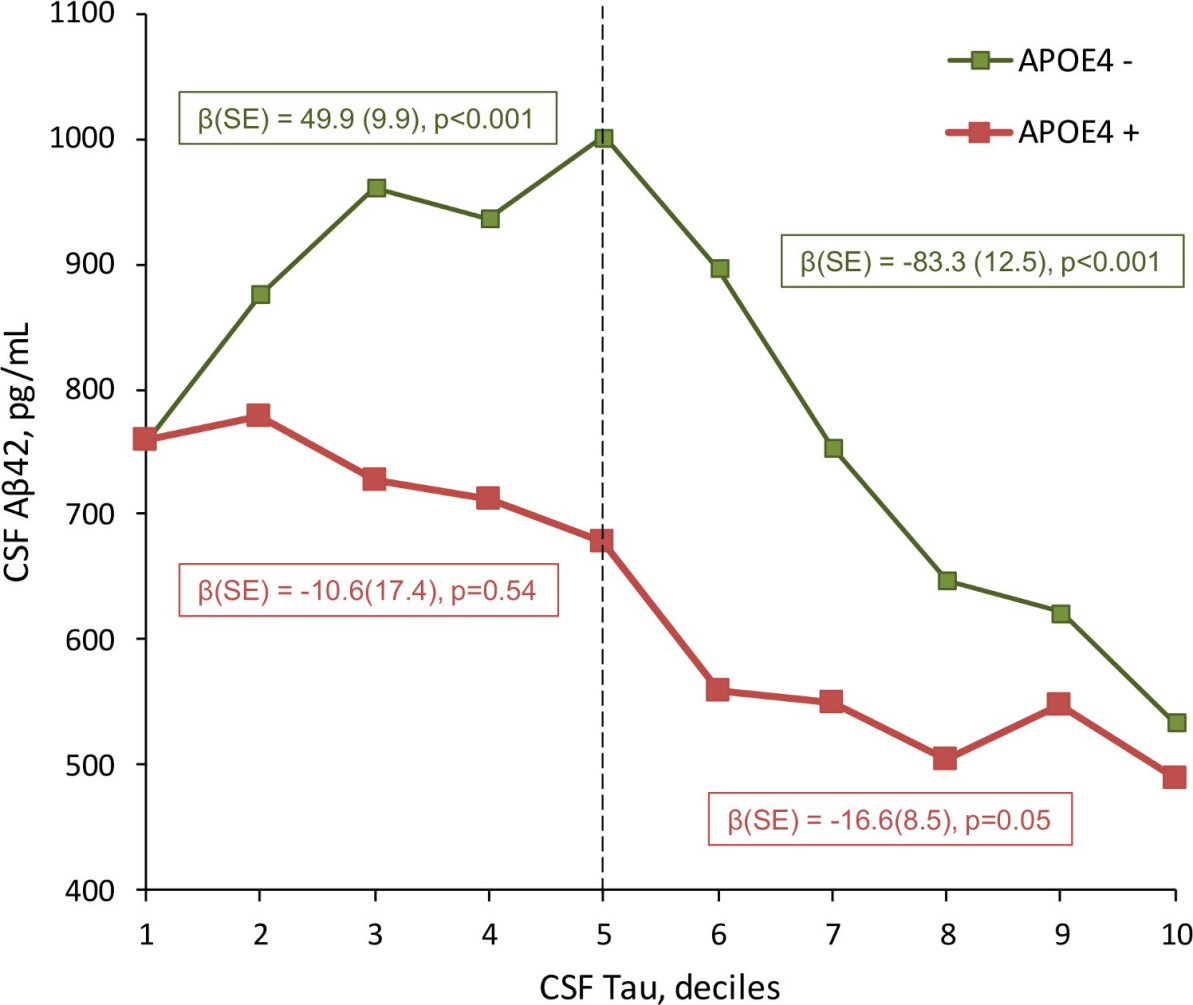
Mean CSF Aβ42 by deciles of CSF Tau, as a function of APOE4 status. Green line: No APOE ε4 allele. Red line: at least one APOE ε4 allele. The linear regression coefficients show analysis between CSF Aβ42 and deciles of CSF Tau are presented by APOE4 status, stratified by the median of CSF Tau.

Fig 2 shows the mean CSF Aβ42 value as a function of deciles of CSF Tau in APOE4 positive and negative participants. In APOE4 negative subjects, the mean value of CSF Aβ42 increased from the first to the 5^th^ decile of CSF Tau (β[SE] = 49.9[9.9], p<0.001), and then decreased for subjects with higher CSF Tau levels (β[SE] = -83.3[12.5], p<0.001). For APOE4 positive subjects, there was no association between CSF Aβ42 and CSF Tau stable until the 5^th^ decile (β[SE] = -10.6[17.4], p = 0.54), and then decreased for those with higher CSF Tau values (β[SE] = -16.6[8.5], p = 0.05).

Finally, Table 3 presents the evolution of profiles among the 70 patients who had 2 lumbar punctures, over a median follow-up of 2.1 years. Of the 23 patients initially classified as A+N- profile, 61% of them remained A+N- at the 2^d^ assessment, and 13% of them evolved to A+N+ profile. For the 14 patients initially classified as A-N+ profile, 50% had the same profile and 36% evolved to an A+N+ profile.

**Table 3:** Change over time in profiles defined by CSF biomarkers profiles in 70 patients with 2 assessments of biomarkers. Profiles are based on CSF Aβ42, CSF Tau, and CSF p-Tau (NIA-AA classification).

	Profile at time 2				
Profile at time 1	A-N-	A+N-	A+N+	A-N+	Total
A-N-	4 (30)	7 (54)	1 (8)	1 (8)	13
A+N-	5 (22)	14 (61)	3 (13)	1 (4)	23
A+N+	0 (0)	2 (10)	16 (80)	2 (10)	20
A-N+	1 (7)	1 (7)	5 (36)	7 (50)	14

## Discussion

We report the following findings on the relationship between CSF Tau and CSF Aβ42 in a large, multicentric cohort of 3565 patients assessed for cognitive disorders:

- We confirmed the previously reported quadratic relationship [22] between these 2 biomarkers in the total population not stratified by biomarker profiles.
- Further analysis revealed that the association between CSF Tau and CSF Aβ42 is strongly dependant on biomarkers profiles. This association was positive in those with a normal profile, no association was found in amyloidosis only subjects, and an inverse association in those with SNAP and AD profiles. The association between CSF Tau and CSF Aβ42 was linear and not quadratic when examined separately in each profile group.
- Analysis of change in biomarkers showed that 36% of those who were initially classified as A-N+ evolved to an A+N+ profile, compared to 13% for those who were initially classified as A+N-.

**Fig 3:**
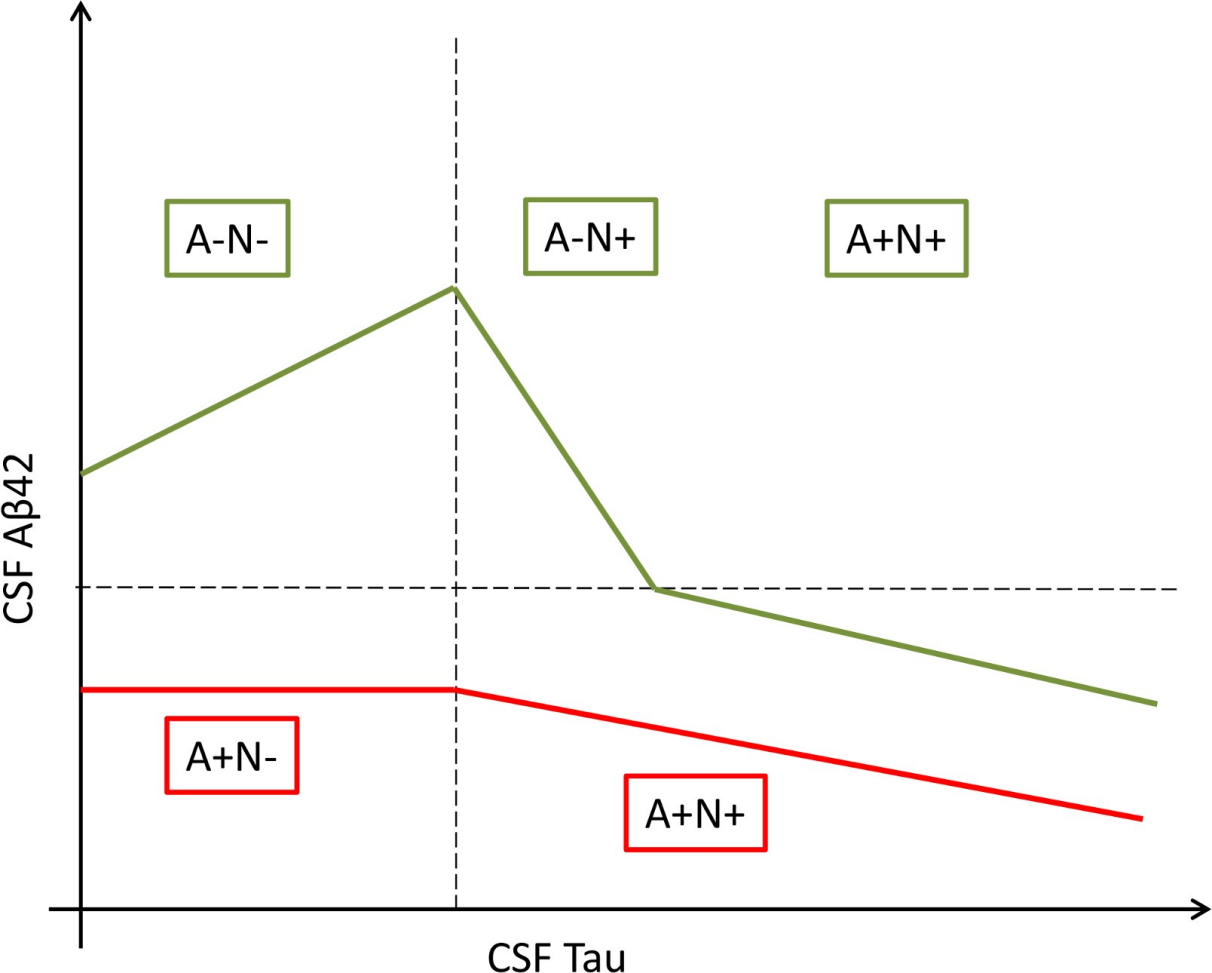
Two distinct patterns: Theoretical framework of the association between CSF Aβ42 and CSF Tau. In green, patients with normal profiles have an increase of CSF Aβ42 followed by a decrease after the progression of tau pathology. In red, patients with isolated low CSF Aβ42 had a stable level before declining.

We propose a theoretical framework to explain these results which suggest two distinct patterns, illustrated by Fig 3. The first pattern is characterized by biomarkers in normal range where level of Aβ42 in the CSF increases with level of CSF Tau reflecting early stage of AD. This stage corresponds to an increase in production and/or decrease in degradation of Aβ42 in the brain which is still being compensated by an increase in its clearance into the CSF. At some point in disease progression systems responsible for clearing Aβ42 from the brain into the CSF become overwhelmed [31] and Aβ42 accumulates in plaques in the brain and CSF Aβ42 levels decrease while CSF Tau continues to increase to abnormal levels, reflecting the progression of tau pathology. Within this pattern, some A-N+ patients evolve to future AD profile as they are simply in the phase that follows the initial increase of CSF Aβ42. This would explain why one third of our patients initially classified as A-N+ finally evolve to an A+N+ profile with biomarkers assessed at the second lumbar puncture. In the second pattern shown in Fig 3, level of CSF Aβ42 remains low and stable during the initial progression of Tau pathology (A+N- patients) to the point where CSF Aβ42 begins to decrease inversely with CSF Tau (A+N+). Interestingly, these two patterns of change in biomarkers reflect that we found in analyses stratified by APOEe4 status; patients without APOE4 allele correspond to the first pattern: initial increase and then decrease of CSF Aβ42 with progression of CSF tau, while patients with at least one APOE4 allele correspond to the second pattern: initial stability (A+N-) and then decrease of CSF Aβ42 with progression of tau pathology.

The kinetics of CSF Aβ42 in very early stages of sporadic AD remains largely unknown. Data from studies on carriers of autosomal-dominant AD mutations,[32, 33] as well as from experimental studies [34], suggest that CSF Aβ42 levels increase before and during the early phase of brain amyloid deposition. In their study on biomarker changes in dominantly inherited Alzheimer’s disease, Bateman et al. reported that CSF Aβ42 levels were higher in persons with autosomal dominant AD 20 to 30 years before the clinical onset of the disease [32], the earliest observed phenomenon associated with AD.

In the recently proposed biological diagnosis of AD based on the A/T/N classification, A-T+ profiles are considered to be non-AD pathologic change [28]. Our findings suggest that this conclusion may need to be tempered in light of our findings that some of these subjects also evolve towards an AD profile. The neuropathological concept of “primary age-related tauopathy” (PART) has recently emerged to characterize the many patients with mainly neurofibrillary pathology and only few or none Aβ deposits [35, 36]. Whether this condition represents an independent disease remains the subject of debate; several authors have suggested that PART belongs to the AD spectrum, with some patients beginning their disease by an initial spreading of tau lesions that precedes Aβ accumulation [4, 37]. CSF biomarkers represents an opportunity to test in-vivo these hypothesis and the fact that one third of our patients initially classified as A-N+ evolved to an A+N+ profile supports this hypothesis. Furthermore, many neuropathological studies have reported that neurofibrillary tangles accumulation is a common feature of ageing [38–40]. Current methods lack sensitivity to detect minimal tangle presence in the entorhinal area and hippocampus, more sensitive methods for tau detection may in the future increase the proportion of A-N+ patients.

This study has several strengths, including its large size which allowed us to cover a wide spectrum of AD pathology, the inclusion of data from 18 memory clinics, and the fact that the analyses of CSF were performed in a single biochemistry department which prevents intersite variability [29]. One of the main limitations is that most of the patients had only one CSF biomarkers assessment, which allows only cross-sectional analysis. We however checked the evolution of biomarkers profiles in 70 patients who had 2 lumbar punctures. Future large scale studies with repeated CSF biomarkers assessments and large duration of follow-up will be helpful to better understand the kinetics of these biomarkers, especially at the early stage of the disease. Another limitation is that the four profiles were defined using CSF biomarkers rather than independent criteria (PET-imaging, neuropathology).

In conclusion, this report describes the relationship between CSF Aβ42 and CSF Tau in a large and multicentric population of patients who were referred to memory clinics for further assessment of cognitive disorders. We found that the nature of the association between these biomarkers was dependent of the biomarker based profile of the patients: positive association in normal profile, negative association in SNAP and AD profiles, lack of association in amyloidosis only subjects. These results plead for an increase of CSF Aβ42 early in the disease, before its decline, especially among non-APOE4 subjects. This pattern may explain why some patients with neurodegeneration only markers convert to AD profiles only over time.

## Supporting information

S1 Table Comparison of linear and quadratic models for the association between CSF Tau (log transformed) and CSF Aβ42. Profiles are based on CSF Aβ42, CSF Tau, and CSF p-Tau (NIA-AA classification).
(DOCX)

## References

[1] Hyman BT, Phelps CH, Beach TG, Bigio EH, Cairns NJ, Carrillo MC, et al National Institute on Aging-Alzheimer's Association guidelines for the neuropathologic assessment of Alzheimer's disease. Alzheimer's & dementia: the journal of the Alzheimer's Association. 2012;8(1):1–13. Epub 2012/01/24. 10.1016/j.jalz.2011.10.007 22265587 PMC3266529

[2] Hardy JA, Higgins GA. Alzheimer's disease: the amyloid cascade hypothesis. Science (New York, NY). 1992;256(5054):184–5. Epub 1992/04/10. . 1566067 10.1126/science.1566067

[3] Reitz C. Alzheimer's disease and the amyloid cascade hypothesis: a critical review. International journal of Alzheimer's disease. 2012;2012:369808 Epub 2012/04/17. 10.1155/2012/369808 22506132 PMC3313573

[4] Braak H, Del Tredici K. Are cases with tau pathology occurring in the absence of Abeta deposits part of the AD-related pathological process? Acta neuropathologica. 2014;128(6):767–72. Epub 2014/11/02. 10.1007/s00401-014-1356-1 . 25359108

[5] Braak H, Del Tredici K. The preclinical phase of the pathological process underlying sporadic Alzheimer's disease. Brain: a journal of neurology. 2015;138(Pt 10):2814–33. Epub 2015/08/19. 10.1093/brain/awv236 . 26283673

[6] Zetterberg H, Lautner R, Skillback T, Rosen C, Shahim P, Mattsson N, et al CSF in Alzheimer's disease. Advances in clinical chemistry. 2014;65:143–72. Epub 2014/09/23. . 25233613 10.1016/b978-0-12-800141-7.00005-x

[7] Blennow K. A Review of Fluid Biomarkers for Alzheimer's Disease: Moving from CSF to Blood. Neurology and therapy. 2017;6(Suppl 1):15–24. Epub 2017/07/25. 10.1007/s40120-017-0073-9 28733960 PMC5520819

[8] Grimmer T, Riemenschneider M, Forstl H, Henriksen G, Klunk WE, Mathis CA, et al Beta amyloid in Alzheimer's disease: increased deposition in brain is reflected in reduced concentration in cerebrospinal fluid. Biological psychiatry. 2009;65(11):927–34. Epub 2009/03/10. 10.1016/j.biopsych.2009.01.027 19268916 PMC2700302

[9] Tapiola T, Alafuzoff I, Herukka SK, Parkkinen L, Hartikainen P, Soininen H, et al Cerebrospinal fluid {beta}-amyloid 42 and tau proteins as biomarkers of Alzheimer-type pathologic changes in the brain. Archives of neurology. 2009;66(3):382–9. Epub 2009/03/11. 10.1001/archneurol.2008.596 . 19273758

[10] Jack CR Jr., Knopman DS, Jagust WJ, Shaw LM, Aisen PS, Weiner MW, et al Hypothetical model of dynamic biomarkers of the Alzheimer's pathological cascade. The Lancet Neurology. 2010;9(1):119–28. Epub 2010/01/20. 10.1016/S1474-4422(09)70299-6 20083042 PMC2819840

[11] Sperling RA, Aisen PS, Beckett LA, Bennett DA, Craft S, Fagan AM, et al Toward defining the preclinical stages of Alzheimer's disease: recommendations from the National Institute on Aging-Alzheimer's Association workgroups on diagnostic guidelines for Alzheimer's disease. Alzheimer's & dementia: the journal of the Alzheimer's Association. 2011;7(3):280–92. Epub 2011/04/26. 10.1016/j.jalz.2011.03.003 21514248 PMC3220946

[12] Jack CR Jr., Knopman DS, Weigand SD, Wiste HJ, Vemuri P, Lowe V, et al An operational approach to National Institute on Aging-Alzheimer's Association criteria for preclinical Alzheimer disease. Annals of neurology. 2012;71(6):765–75. Epub 2012/04/11. 10.1002/ana.22628 22488240 PMC3586223

[13] Jack CR Jr., Knopman DS, Chetelat G, Dickson D, Fagan AM, Frisoni GB, et al Suspected non-Alzheimer disease pathophysiology—concept and controversy. Nature reviews Neurology. 2016;12(2):117–24. Epub 2016/01/20. 10.1038/nrneurol.2015.251 26782335 PMC4784257

[14] Caroli A, Prestia A, Galluzzi S, Ferrari C, van der Flier WM, Ossenkoppele R, et al Mild cognitive impairment with suspected nonamyloid pathology (SNAP): Prediction of progression. Neurology. 2015;84(5):508–15. Epub 2015/01/09. 10.1212/WNL.0000000000001209 25568301 PMC4336071

[15] Prestia A, Caroli A, van der Flier WM, Ossenkoppele R, Van Berckel B, Barkhof F, et al Prediction of dementia in MCI patients based on core diagnostic markers for Alzheimer disease. Neurology. 2013;80(11):1048–56. Epub 2013/02/08. 10.1212/WNL.0b013e3182872830 . 23390179

[16] Vos SJ, Verhey F, Frolich L, Kornhuber J, Wiltfang J, Maier W, et al Prevalence and prognosis of Alzheimer's disease at the mild cognitive impairment stage. Brain: a journal of neurology. 2015;138(Pt 5):1327–38. Epub 2015/02/20. 10.1093/brain/awv029 25693589 PMC5013930

[17] Chung JK, Plitman E, Nakajima S, Caravaggio F, Shinagawa S, Iwata Y, et al The Effects of Cortical Hypometabolism and Hippocampal Atrophy on Clinical Trajectories in Mild Cognitive Impairment with Suspected Non-Alzheimer's Pathology: A Brief Report. Journal of Alzheimer's disease: JAD. 2017;60(2):341–7. Epub 2017/08/23. 10.3233/JAD-170098 . 28826178

[18] Jack CR Jr., Bennett DA, Blennow K, Carrillo MC, Feldman HH, Frisoni GB, et al A/T/N: An unbiased descriptive classification scheme for Alzheimer disease biomarkers. Neurology. 2016;87(5):539–47. Epub 2016/07/03. 10.1212/WNL.0000000000002923 27371494 PMC4970664

[19] Burnham SC, Bourgeat P, Dore V, Savage G, Brown B, Laws S, et al Clinical and cognitive trajectories in cognitively healthy elderly individuals with suspected non-Alzheimer's disease pathophysiology (SNAP) or Alzheimer's disease pathology: a longitudinal study. Lancet Neurol. 2016;15(10):1044–53. Epub 2016/07/28. 10.1016/S1474-4422(16)30125-9 . 27450471

[20] Gordon BA, Blazey T, Su Y, Fagan AM, Holtzman DM, Morris JC, et al Longitudinal beta-Amyloid Deposition and Hippocampal Volume in Preclinical Alzheimer Disease and Suspected Non-Alzheimer Disease Pathophysiology. JAMA neurology. 2016;73(10):1192–200. Epub 2016/08/23. 10.1001/jamaneurol.2016.2642 27548756 PMC5237381

[21] Mormino EC, Papp KV, Rentz DM, Schultz AP, LaPoint M, Amariglio R, et al Heterogeneity in Suspected Non-Alzheimer Disease Pathophysiology Among Clinically Normal Older Individuals. JAMA neurology. 2016;73(10):1185–91. Epub 2016/08/23. 10.1001/jamaneurol.2016.2237 27548655 PMC5266522

[22] de Leon MJ, Pirraglia E, Osorio RS, Glodzik L, Saint-Louis L, Kim HJ, et al The nonlinear relationship between cerebrospinal fluid Abeta42 and tau in preclinical Alzheimer's disease. PloS one. 2018;13(2):e0191240 Epub 2018/02/08. 10.1371/journal.pone.0191240 29415068 PMC5802432

[23] Mouton-Liger F, Wallon D, Troussiere AC, Yatimi R, Dumurgier J, Magnin E, et al Impact of cerebro-spinal fluid biomarkers of Alzheimer's disease in clinical practice: a multicentric study. Journal of neurology. 2014;261(1):144–51. Epub 2013/10/29. 10.1007/s00415-013-7160-3 . 24162039

[24] Troussiere AC, Wallon D, Mouton-Liger F, Yatimi R, Robert P, Hugon J, et al Who needs cerebrospinal biomarkers? A national survey in clinical practice. Journal of Alzheimer's disease: JAD. 2014;40(4):857–61. Epub 2014/03/01. 10.3233/JAD-132672 . 24577469

[25] Paquet C, Bouaziz-Amar E, Cognat E, Volpe-Gillot L, Haddad V, Mahieux F, et al Distribution of Cerebrospinal Fluid Biomarker Profiles in Patients Explored for Cognitive Disorders. Journal of Alzheimer's disease: JAD. 2018 Epub 2018/07/04. 10.3233/jad-180240 . 29966201

[26] Boumenir A, Cognat E, Sabia S, Hourregue C, Lilamand M, Dugravot A, et al CSF level of β-amyloid peptide predicts mortality in Alzheimer’s disease. Alzheimer's Research & Therapy. 2019;11(1):29 10.1186/s13195-019-0481-4 30922415 PMC6440001

[27] Mattsson N, Andreasson U, Persson S, Carrillo MC, Collins S, Chalbot S, et al CSF biomarker variability in the Alzheimer's Association quality control program. Alzheimer's & dementia: the journal of the Alzheimer's Association. 2013;9(3):251–61. Epub 2013/04/30. 10.1016/j.jalz.2013.01.010 23622690 PMC3707386

[28] Jack CR Jr., Bennett DA, Blennow K, Carrillo MC, Dunn B, Haeberlein SB, et al NIA-AA Research Framework: Toward a biological definition of Alzheimer's disease. Alzheimer's & dementia: the journal of the Alzheimer's Association. 2018;14(4):535–62. Epub 2018/04/15. 10.1016/j.jalz.2018.02.018 29653606 PMC5958625

[29] Dumurgier J, Vercruysse O, Paquet C, Bombois S, Chaulet C, Laplanche JL, et al Intersite variability of CSF Alzheimer's disease biomarkers in clinical setting. Alzheimer's & dementia: the journal of the Alzheimer's Association. 2013;9(4):406–13. Epub 2012/11/13. 10.1016/j.jalz.2012.06.006 . 23141384

[30] Yamaoka K, Nakagawa T, Uno T. Application of Akaike's information criterion (AIC) in the evaluation of linear pharmacokinetic equations. Journal of pharmacokinetics and biopharmaceutics. 1978;6(2):165–75. Epub 1978/04/01. . 671222 10.1007/BF01117450

[31] Tarasoff-Conway JM, Carare RO, Osorio RS, Glodzik L, Butler T, Fieremans E, et al Clearance systems in the brain-implications for Alzheimer disease. Nature reviews Neurology. 2015;11(8):457–70. Epub 2015/07/22. 10.1038/nrneurol.2015.119 26195256 PMC4694579

[32] Bateman RJ, Xiong C, Benzinger TL, Fagan AM, Goate A, Fox NC, et al Clinical and biomarker changes in dominantly inherited Alzheimer's disease. The New England journal of medicine. 2012;367(9):795–804. Epub 2012/07/13. 10.1056/NEJMoa1202753 22784036 PMC3474597

[33] Fagan AM, Xiong C, Jasielec MS, Bateman RJ, Goate AM, Benzinger TL, et al Longitudinal change in CSF biomarkers in autosomal-dominant Alzheimer's disease. Science translational medicine. 2014;6(226):226ra30 Epub 2014/03/07. 10.1126/scitranslmed.3007901 24598588 PMC4038930

[34] Maia LF, Kaeser SA, Reichwald J, Lambert M, Obermuller U, Schelle J, et al Increased CSF Abeta during the very early phase of cerebral Abeta deposition in mouse models. EMBO molecular medicine. 2015;7(7):895–903. Epub 2015/05/17. 10.15252/emmm.201505026 25978969 PMC4520655

[35] Crary JF, Trojanowski JQ, Schneider JA, Abisambra JF, Abner EL, Alafuzoff I, et al Primary age-related tauopathy (PART): a common pathology associated with human aging. Acta neuropathologica. 2014;128(6):755–66. Epub 2014/10/29. 10.1007/s00401-014-1349-0 25348064 PMC4257842

[36] Jellinger KA, Alafuzoff I, Attems J, Beach TG, Cairns NJ, Crary JF, et al PART, a distinct tauopathy, different from classical sporadic Alzheimer disease. Acta neuropathologica. 2015;129(5):757–62. Epub 2015/03/18. 10.1007/s00401-015-1407-2 25778618 PMC4534004

[37] Duyckaerts C, Braak H, Brion JP, Buee L, Del Tredici K, Goedert M, et al PART is part of Alzheimer disease. Acta neuropathologica. 2015;129(5):749–56. Epub 2015/01/30. 10.1007/s00401-015-1390-7 25628035 PMC4405349

[38] Bouras C, Hof PR, Giannakopoulos P, Michel JP, Morrison JH. Regional distribution of neurofibrillary tangles and senile plaques in the cerebral cortex of elderly patients: a quantitative evaluation of a one-year autopsy population from a geriatric hospital. Cerebral cortex (New York, NY: 1991). 1994;4(2):138–50. Epub 1994/03/01. . 8038565 10.1093/cercor/4.2.138

[39] Hyman BT, Gomez-Isla T. The natural history of Alzheimer neurofibrillary tangles and amyloid deposits. Neurobiology of aging. 1997;18(4):386–7; discussion 9–92. Epub 1997/07/01. . 9330968 10.1016/s0197-4580(97)00054-7

[40] Tsartsalis S, Xekardaki A, Hof PR, Kovari E, Bouras C. Early Alzheimer-type lesions in cognitively normal subjects. Neurobiology of aging. 2018;62:34–44. Epub 2017/11/07. 10.1016/j.neurobiolaging.2017.10.002 29107845 PMC5743763

