# Supplementary material for: Biomarker profiles of Alzheimer’s disease and dynamic of the association between cerebrospinal fluid levels of β-amyloid peptide and tau
Source: PLoS One. 2019 May 14;14(5):e0217026. doi: 10.1371/journal.pone.0217026 (PMC6516653; doi:10.1371/journal.pone.0217026)
Supplement: S1 Table — Profiles are based on CSF Aβ42, CSF Tau, and CSF p-Tau (NIA-AA classification). (DOCX) [file pone.0217026.s001.docx]

**S1 Table. Comparison of linear and quadratic models for the association between CSF Tau (log transformed) and CSF Aβ42.** Profiles are based on CSF Aβ42, CSF Tau, and CSF p-Tau (NIA-AA classification).

|  |  |  |  |
| --- | --- | --- | --- |
|  | Akaike information criterion (AIC) | | |
| Population | Linear model | Quadratic model | Delta AIC^a^ |
| Overall | -2887 | -2930 | 43 |
| Profiles |  |  |  |
| A-N- | -2336 | -2334 | -2 |
| A+N- | -1368 | -1366 | -2 |
| A+N+ | -1971 | -1969 | -2 |
| A-N+ | -869 | -1877 | 8 |
|  | | | |

^a^A positive value represents a superiority of the quadratic model.
